# Consensus on the Cosmetic Use of a Novel Botulinum Neurotoxin Type A Product (NEWLUX^®^) for Facial Expression Muscles: 2024 Guidelines and Discussions by Korean Experts

**DOI:** 10.3390/toxins17020061

**Published:** 2025-01-29

**Authors:** Nark-Kyoung Rho, Gee Young Bae, Moon Seop Choi, Woon-Kyong Chung, Hoon-Young Kim, Hyoung Moon Kim, Hong-Ki Lee, Yong Woo Lee, Wook Oh, Wu-Chul Song

**Affiliations:** 1 Leaders Aesthetic Laser and Cosmetic Surgery Center, Seoul 06014, Republic of Korea; 2 Rose Dermatology Clinic, Seongnam 13555, Republic of Korea; 3 Grace Plastic Surgery, Seoul 06524, Republic of Korea; 4 You&Chung Skin Clinic, Seoul 06062, Republic of Korea; shinyskinned@daum.net; 5 Pygmalion Clinic, Seoul 06070, Republic of Korea; parnate@naver.com; 6 Maylin Clinic, Goyang 10391, Republic of Korea; md.mac12@gmail.com; 7 Image Plastic Surgery Clinic, Seoul 06180, Republic of Korea; cyberhg@naver.com; 8 LIKE Plastic Surgery Clinic, Seoul 06612, Republic of Korea; ywlee82@hanmail.net; 9 Maylin Clinic the Hyundai, Seoul 07335, Republic of Korea; feelclinic@naver.com; 10 Beautiful Stroy Clinic, Seoul 06044, Republic of Korea; sikkakki@gmail.com

**Keywords:** botulinum toxin type A, consensus, expert opinions, mimetic muscles, practice guidelines

## Abstract

Background: Botulinum neurotoxin type A (BoNTA) is widely used in aesthetic facial rejuvenation medicine. The exponential growth in using BoNTA for cosmetic purposes in Korea necessitates an update to the existing practice guidelines, building upon the consensus established by Korean experts in 2013. Aims: This work aims to provide an updated Korean consensus guideline for the safe and effective use of a novel BoNTA product (NEWLUX) for facial expression muscles. Methods: A panel of ten Korean experts in dermatology, plastic surgery, aesthetic medicine, and clinical anatomy convened in February 2024. They reviewed prior guidelines, including the 2013 Korean consensus, and shared their expertise on dosage, injection techniques, and potential complications associated with the use of the novel BoNTA product for facial expression muscles. The recommendations aimed to guide the best practices of the average aesthetic healthcare professional. Results: The panel reached a consensus on various aspects of using the BoNTA product, including recommended injection sites, dosages, and techniques for different aesthetic conditions caused by facial expression muscles. The resulting guideline emphasizes safety and efficacy, with recommendations based on the collective experience of the expert panel. Conclusion: This updated consensus guideline provides practical recommendations from Korean experts on the use of the novel BoNTA product for facial expression muscles. The guidelines will ensure safe and effective treatments while reflecting the latest advancements in the field.

## 1. Introduction

Botulinum neurotoxin type A (BoNTA) has become a fundamental element in contemporary aesthetic medicine, extending its applications beyond the initially approved indications. The expanding reliance on this neurotoxin for cosmetic procedures in Korea reflects the increasing emphasis on non-surgical facial enhancement [1]. In 2013, a panel of Korean experts established a consensus on the cosmetic use of BoNTA [2], which became a standard reference for aesthetic medicine and surgery in Korea. However, over the past decade, the need to update this guideline has arisen due to the significant research progress made in cosmetic BoNTA applications and the availability of new BoNTA products on the market. Given the distinct properties of different BoNTA formulations, using a specific BoNTA product according to its specific recommendations is crucial, as supported by clinical evidence and practical experience [3]. This paper provides comprehensive and up-to-date expert recommendations to guide aesthetic healthcare professionals in safely and effectively using BoNTA for a wide range of aesthetic concerns related to facial expression muscles, focusing on the clinical use of a newly developed BoNTA formulation.

## 2. Results and Discussion

### 2.1. Glabellar Frown Lines

BoNTA is a well-recognized and widely utilized treatment for managing dynamic glabellar lines in Korea [4], as evidenced by a recent survey indicating that Korean dermatologists most frequently target the glabella region for the cosmetic application of this neurotoxin [1]. However, side effects, including ptosis and altered eyebrow positioning, may arise after injection due to insufficient knowledge of the detailed facial anatomy of the glabella area [5].

#### 2.1.1. Anatomy and Patient Selection

The corrugator supercilii and procerus muscles are the primary muscles responsible for glabellar frown lines. The corrugator supercilii muscle is located at the medial portion of the eyebrow, while the procerus muscle extends from the nasal bridge to the glabella. Additionally, the superomedial fibers of the orbicularis oculi, known as the depressor supercilii [5], are interconnected with the corrugator and procerus muscles. These glabellar muscles are not independent; rather, they are closely interconnected. As a result, BoNTA injected into the corrugator supercilii may affect the nearby muscles [5].

Ethnic considerations: Studies have found ethnic differences in the size and shape of the corrugator muscles responsible for glabellar frown lines. For example, researchers observed that the corrugators were significantly smaller in 18% of Japanese cadavers compared to Caucasian cadavers [6].

Patient selection: Patients of younger age with greater skin elasticity often demonstrate more favorable responses to BoNTA injections for managing glabellar lines. However, the efficacy of this treatment is not necessarily constrained by a patient’s age, as beneficial outcomes can be achieved across various age groups.

#### 2.1.2. Recommended Standard Injection Technique

**Figure 1:**
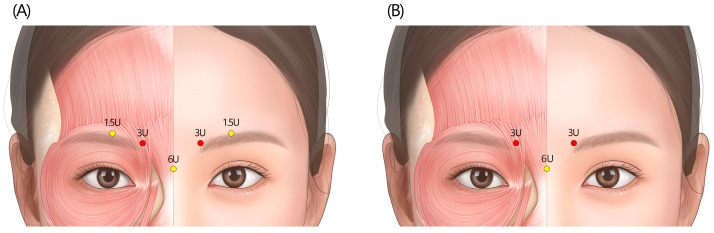
The recommended injection techniques for glabella frown lines. (**A**) The standard “five-point injection” technique is recommended for treating moderate glabellar frown lines. (**B**) The “three-point injection” technique [2] can be used for patients with limited corrugator muscle contraction, focusing the injection only on the corrugator heads and sparing the corrugator tails. U = unit(s); yellow circle = subcutaneous injection; red circle = intramuscular injection.

Inject BoNTA into the medial and lateral parts of the corrugator muscle, followed by an injection into the center of the procerus muscle (the “five-point injection” technique: Figure 1A). An intramuscular injection is recommended for the corrugator head, while a subdermal placement is preferable for the corrugator tail and the procerus. Proceed with the injections slowly to prevent the spread of the toxin. Avoid injecting too close or deep to the orbital rim, which may result in eyelid ptosis. It is also recommended that the frontal notch be pressed with the fingers during injection to avoid the inflow of the toxin into the orbital cavity. When the extent of the corrugator contraction is limited, only the corrugator head portion may be injected (the “three-point injection” technique: Figure 1B). A typical protocol for Korean female patients involves injecting 3 BoNTA units (U) into each corrugator head, 1.5 U into each corrugator tail, and 6 U into the procerus to treat moderate glabellar lines.

#### 2.1.3. Changes from the 2013 Guideline

The panel recommended the five-point injection approach as the standard technique, moving away from the more conservative, three-point injection technique suggested in the 2013 guideline. The panel’s consensus was that the five-point injection technique provides a more comprehensive and reliable treatment of glabellar frown lines. Targeting the corrugator muscle’s head and tail may lead to more complete paralysis, potentially improving overall efficacy [5]. The panel acknowledged that some patients may need higher doses, up to 20 units, to achieve the desired result, compared to the more conservative 12-unit recommendation in the previous guideline. The rationale for using higher doses is based on recent findings that a 2–4-fold increase in the BoNTA dose for glabella leads to a longer duration of clinical effects while maintaining patient satisfaction and a natural appearance [7].

#### 2.1.4. Expert’s Opinions

Anatomical diversity: A recent Korean cadaver study [8] revealed diverse fiber extensions and varied trajectories and lengths of the corrugator supercilii muscle, which extends towards the upper face and midface. Additionally, the lower fibers of the corrugator supercilii muscle were found to connect to important structures like the orbicularis oculi, the malaris, and the superficial musculoaponeurotic system. These findings emphasize the importance of a precise BoNTA injection technique to avoid unintended effects, as the anatomy must be carefully considered.

Age and gender: The panel agreed that a patient’s age and gender should be factored into the selection of injection technique and dosage. Younger female patients, who typically present with less severe glabellar lines and weaker muscle activity, could benefit from the three-point injection approach [2]. In contrast, aged patients, regardless of gender, often require the five-point injection technique due to the increased prominence of glabellar lines and potentially stronger muscle activity. Additionally, male patients, who generally exhibit stronger corrugator muscles than their female counterparts, may require higher dosages to achieve comparable results.

**Figure 2:**
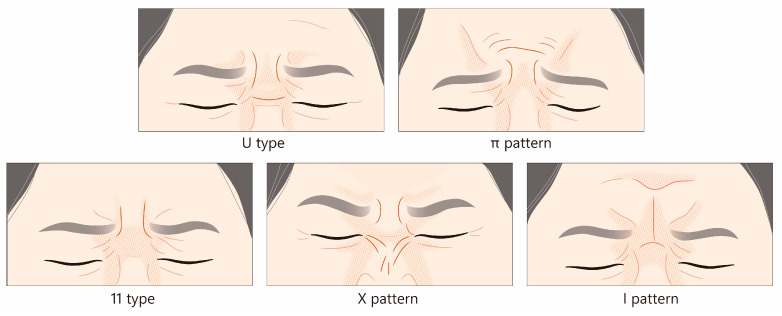
Five common glabella wrinkle patterns in Koreans, as categorized by a panel of experienced Korean dermatologists [9]. This classification could help identify the key muscles contributing to each wrinkle pattern, guiding the targeted application of botulinum toxin.

Identifying the contraction pattern: Understanding individual variation in muscle use is key to achieving natural, personalized results when treating glabellar wrinkles with botulinum toxin. Classifying each patient’s wrinkle patterns helps guide the dosage to target the specific muscle regions contributing most to their wrinkle formation [9]. The panel emphasized meticulously observing each patient’s muscle movement patterns during frowning (Figure 2). Relying solely on standard anatomical landmarks might not suffice for optimal outcomes. Some experts suggested that identifying the specific locations of “eyebrow dimples” formed during frowning can provide a more precise guide for deciding the injection point.

On the other hand, a recent anatomical study has found that glabellar contraction patterns do not strongly correlate with the underlying muscle anatomy, suggesting that relying solely on contraction patterns to guide BoNTA treatment algorithms may not always be clinically optimal [10]. In this context, it is paramount to closely evaluate the patient’s treatment response after the initial BoNTA injections. The panel strongly emphasized that using ultrasound imaging during the injection procedure can substantially aid in overcoming anatomical variations and enhance the overall efficacy of glabellar injections, a rapidly growing application in aesthetic medicine and surgery [11].

The “Eyebrow Illusion”: The panel cautioned against being misled by the resting position of a patient’s eyebrows, particularly in individuals with eyebrow makeup or tattoos. Patients with “drawn” eyebrows characterized by high arches can make an illusion when identifying the location of corrugator tails. This could lead to injecting BoNTA too superiorly and unintentionally suppressing the lower part of the frontalis muscle. The panel stressed the importance of carefully assessing the “real” eyebrow position concerning muscle activity to avoid such complications. Special care should also be taken when treating patients with a high insertion of the corrugator supercilii muscle, as they may be at an increased risk of developing brow ptosis following the glabella injection.

Benefits of subdermal injections: In the context of safety, the panel concurred that subdermal injections have advantages when treating the glabellar complex with BoNTA. Although deep injection is required to paralyze the corrugator supercilli completely, injecting too deeply increases the risk of diffusion to the surrounding structures, potentially leading to eyelid ptosis. The panel suggested that the injections can target the subcutaneous tissue or, at most, the superficial layers of the muscle to minimize the risk of diffusion and optimize the precision of treatment.

Further anatomical considerations for the procerus muscle: Unlike corrugator injections, injections targeting the procerus muscle pose a relatively low risk of eyelid ptosis. Given that the procerus originates from both superficial and deep layers [12], a combination technique involving subdermal and intramuscular injections into the procerus muscle may help achieve optimal outcomes for certain patients. Another anatomical consideration regarding the procerus injection is that while it is often described as a single pyramidal muscle, detailed dissections have shown that the procerus actually consists of bilateral muscle bundles [13]. These bilateral components originate from the nasal bones on each side and are inserted into the skin of the glabella. In patients with a divided procerus muscle, it may be more appropriate to administer the injection at two points rather than a single midline injection.

Forehead injections: The “quizzical” raised eyebrows complication is not a direct result of treating the glabella area but rather due to the close anatomical connection between the corrugator and frontalis muscles. Injecting the glabella can impact the adjacent frontalis muscle, particularly in patients with brow ptosis or strong brow activity, leading to an elevation of the eyebrow tails. While the recommended guideline focuses on the procerus and corrugator supercilii muscles for the glabellar lines, the panel suggested additional injections into the inferolateral part of the frontalis muscle to prevent this complication in certain patients.

**Figure 3:**
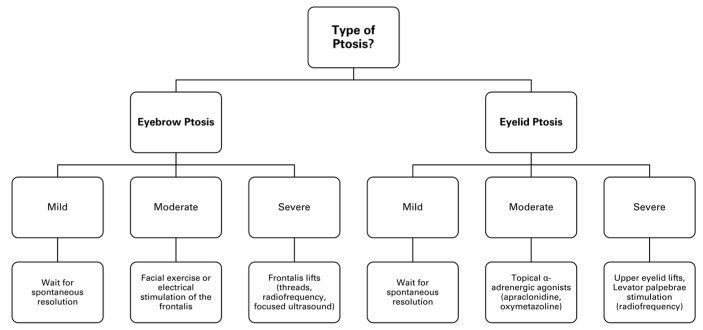
A flow chart outlining practical solutions for BoNTA-related ptosis.

Management of ptosis: A flow chart illustrating practical solutions for BoNTA-related ptosis is provided in Figure 3 to serve as a quick reference guide for clinicians.

### 2.2. Horizontal Forehead Lines

The frontalis muscle plays a pivotal role in forehead aesthetics. As the primary muscle responsible for forehead expressions, it enables a range of movements, notably raising the eyebrows. The activity of this muscle leads to the formation of horizontal lines across the forehead. These lines are not merely a universal sign of aging but vary significantly in their appearance and depth, influenced by individual factors such as genetic predisposition, lifestyle, and habitual facial expressions [14]. BoNTA injections are a widely accepted non-surgical approach for treating dynamic forehead wrinkles, representing the second most common indication for BoNTA injections among Korean dermatologists [1].

#### 2.2.1. Anatomy and Patient Selection

The occipitofrontalis muscle, situated beneath the forehead and occipital region, is divided into the frontal and occipital bellies. The frontal belly, commonly referred to as the frontalis muscle, originates from the galea aponeurosis; interacts with adjacent muscles such as the corrugator supercilii, procerus, and orbicularis oculi; and attaches to the skin above the superciliary arch. The muscle belly is characterized by a bifurcated, V-shaped configuration, with the superomedial portion comprising aponeurosis [15]. These anatomical features exhibit a high degree of individual variation [16].

Ethnic considerations: Research suggests that Europeans generally exhibit more pronounced facial expressions, particularly in the forehead region, compared to Asians [17]. This finding is supported by a study that compared wrinkle scores related to facial expressions between Caucasian and Japanese women [18]. These results indicate that Caucasians may have higher frontalis muscle contractility than Asians. Ethnic variations exist in the relative length of the forehead. For instance, Korean women often exhibit relatively longer foreheads than their white North American counterparts [19].

Patient selection: Younger patients with high skin elasticity and no pre-existing eyelid or brow drooping usually experience better treatment outcomes and minimal post-injection discomfort. Conversely, older patients may face a higher risk of complications like eyelid/eyebrow ptosis, especially if they have a weakened orbital septum.

#### 2.2.2. Recommended Standard Injection Technique

**Figure 4:**
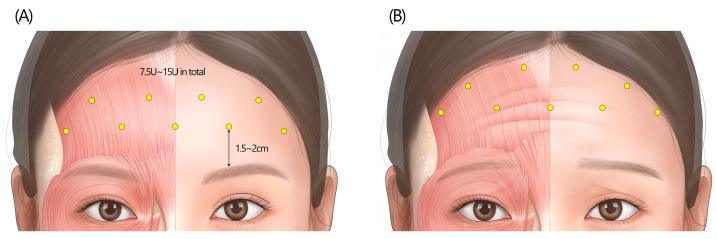
The recommended injection techniques for horizontal forehead lines. (**A**) The standard “zigzag” pattern injections, sparing approximately 1.5 cm above the brow. (**B**) A variation in the forehead injection technique, extending injections to the uppermost portion of the forehead in cases where patients exhibit pronounced muscle contraction in the upper portion of the frontalis. U = unit(s); yellow circle = subcutaneous injection.

Inject BoNTA at 8–10 sites across the forehead in a “zigzag” fashion, approximately 1.5–2 cm above the brow (Figure 4A). Injections should target the central and lateral portions of the frontalis muscle, with typical doses ranging from 7.5 to 15 U in total. Injections are recommended to be administered in the superficial layers, preferably within the subcutaneous adipose tissue. The doses should be adjusted based on the patient’s age, muscle activity, and desired outcome. For instance, the starting dosage can be as low as 5–6 U for young female patients seeking a natural-looking facial appearance.

#### 2.2.3. Changes from the 2013 Guideline

The current guideline highlights the need for a more customized approach, moving away from the rigid, predetermined injection points and dosages outlined in the 2013 guideline. The panel emphasized that forehead anatomy and muscle activity can vary significantly from one individual to another and that a “one-size-fits-all” approach may not be optimal.

#### 2.2.4. Expert’s Opinions

Individualized approaches: The expert panel unanimously emphasized that achieving a true consensus on specific injection techniques and dosages for BoNTA injections in the forehead is inherently challenging. This challenge stems from the wide range of individual variations in patient anatomy, aesthetic goals, and responses to treatment. The panel strongly advocated developing individualized treatment plans beyond the present guideline based on a comprehensive assessment of each patient’s unique characteristics. This may involve considerations such as the depth and pattern of forehead wrinkles, the patient’s age, gender, and skin laxity, as well as the clinician’s experience and preference for specific BoNTA formulations. The panel generally agreed that the fundamental principles of injection point location remain consistent across genders. In contrast, dosage should be adjusted to achieve comparable results between the males and the females.

Superficial approaches: Although the term “intramuscular” is commonly used to describe injections into the frontalis muscle, the panel acknowledged that the actual injection depth should be superficial. Administering BoNTA in the subdermal plane allows the injected toxin to be easily distributed to the frontalis muscle, producing effects comparable to direct intramuscular injection [15]. Research has shown that intradermal administration of BoNTA in the forehead achieves wrinkle reduction outcomes similar to intramuscular injection, while the intradermal approach preserves the natural brow position [20].

**Figure 5:**
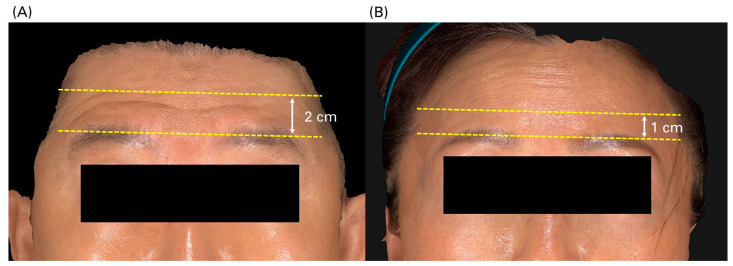
Variations in the “no-injection zone” for treating forehead wrinkles with botulinum neurotoxin, based on different anatomical scenarios. (**A**) A vertical distance of 2 cm was left free from injections, as the patient exhibited greater lower frontalis engagement when raising the eyebrows. (**B**) The no-injection zone was set 1 cm from the eyebrows, as the patient predominantly used the upper part of the frontalis during eyebrow elevation.

Determining the “no-injection” zone: A significant portion of the panel discussion centered on establishing a clearly defined “no-injection” zone to minimize the risk of brow ptosis. While many guidelines recommend avoiding injections within 1.5 cm above the eyebrow, the panel emphasized that this distance should not be considered absolute. The appropriate no-injection zone should be determined based on the patient’s reliance on the frontalis muscle to open the eyes. Patients with greater lower frontalis engagement necessitate a more extensive zone to spare, sometimes exceeding 2 cm (Figure 5). To ascertain the appropriate no-injection area, clinicians should instruct patients to fully open their eyes to assess the degree of frontalis compensation. The initial dosage of BoNTA should be tailored according to the patient’s ability to open the eyes without excessive frontalis muscle dependence.

Upper frontalis injections: Recent research has found that 40% of Korean study participants exhibited bidirectional forehead skin displacement, with the lower portion moving upward and the upper portion moving downward [21]. In cases where patients exhibit pronounced muscle contraction in the upper part of the frontalis muscle, the panel recommended locating injections to a more superior part of the frontalis (Figure 4B). This targeted approach helps to relax the entire muscle complex, ensuring a more harmonious reduction in forehead wrinkles and a more balanced aesthetic outcome.

Impact of the brow position: Clinicians should employ appropriate techniques to preserve a harmonious eyebrow positioning. Patients with naturally high-arched or elevated brows warrant cautious treatment, as BoNTA may further exaggerate brow elevation, resulting in an unnatural aesthetic outcome. Conversely, overly lateral injections in individuals with limited lateral frontalis attachment can precipitate brow ptosis [16].

### 2.3. Lateral Canthal Lines (“Crow’s Feet”) and Lower Eyelid Lines

Over time, the dynamic lines around the eyes in young individuals, formed from smiling and squinting, mature into permanent, stable wrinkles known as crow’s feet in the lateral canthal region by the mid-to-late thirties [22]. This age-related change often leads to patient requests for rejuvenating treatments. BoNTA is an effective and safe intervention for managing periorbital wrinkles, including crow’s feet and lower eyelid wrinkles.

#### 2.3.1. Anatomy and Patient Selection

The orbicularis oculi muscle surrounds the eyes and is responsible for closing the eyelids. It is divided into two main parts: the orbital and palpebral portions. The orbital portion plays a pivotal role in forming crow’s feet. This portion originates from the supraorbital and infraorbital margins of the bony orbital rim and extends to the medial and lateral canthal tendon, skin, and fibers of the frontalis, procerus, and corrugator supercilii muscles [23].

Ethnic considerations: Research has shown that the orbicularis oculi muscle fibers are smaller in Asian individuals than in Europeans [24]. Additionally, Koreans tend to have thicker facial skin [25], which may contribute to less prominent periorbital wrinkles, including crow’s feet.

Patient selection: Ideal candidates are typically mature adults showing signs of aging but still have healthy skin with moderate elasticity.

#### 2.3.2. Recommended Standard Injection Technique

**Figure 6:**
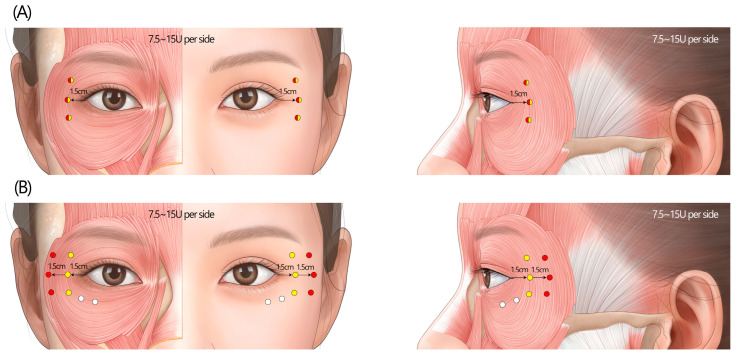
The recommended injection techniques for lateral canthal lines. (**A**) The standard 3- or 5-point injections, along the line 1.5 cm lateral to the outer canthus. (**B**) The “2-line injection” approach involves administering additional injections further laterally to target wrinkles that extend beyond the typical treatment area or are associated with specific muscle movement patterns. Intradermal injections into the lower eyelid skin may prove advantageous, yet the dosage should be confined to 1–2 units per side. U = unit(s); white circle = intradermal injection; yellow circle = subcutaneous injection; red circle = intramuscular injection.

Inject BoNTA at 3–5 sites on the lateral aspect of the orbital portion of the orbicularis oculi muscle, approximately 1.5 cm lateral to the outer canthus (Figure 6A). Recommended doses typically range from 7.5 to 15 U per side (a total of 15–30 U). Injection depth should be superficial, targeting the muscle or subcutaneous fat layer. Stretching the skin during injection is helpful to reduce the chance of bruising. Supplementing one or two intradermal injections into the lower eyelid skin may be beneficial, but the dosage should be restricted.

#### 2.3.3. Changes from the 2013 Guideline

While the panel continues to endorse the three-point injection technique for younger patients, it acknowledges the potential benefits of increasing the number of injection sites for older individuals with more pronounced wrinkles (Figure 6B). Furthermore, the updated guidelines move away from the intradermal injection approach recommended in 2013, instead suggesting the administration of the toxin into the muscle or subcutaneous fat layer, which may lead to more consistent and long-lasting therapeutic effects. Importantly, the panel emphasizes the need for individualized dosing based on the patient’s unique anatomical features, wrinkle severity, and desired aesthetic goals.

#### 2.3.4. Expert Opinions

Subcutaneous vs. intramuscular injections: Some panel members preferred subdermal injections, citing the potential for a more superficial effect and a reduced risk of muscle weakness. Conversely, other panel members favored intramuscular injections, highlighting their effectiveness in achieving more pronounced muscle relaxation, particularly into the thicker portion of the orbicularis oculi muscle, which has an ample subcutaneous layer located approximately 3.0 cm lateral to the lateral canthus [26].

Dilution ratio: A more dilute BoNTA preparation with a larger injection volume can result in a broader distribution, reaching more distant neuromuscular junctions [27]. The panel recommended employing a smaller injection volume with a lower dilution ratio, e.g., 2.5 U in 0.05 mL of reconstituted BoNTA (2 mL/100 U dilution), as this approach may help minimize the spread of the toxin and mitigate the risk of undesirable side effects. This is particularly important when treating areas with thinner muscles or when injecting near the canthus.

Gender differences: Anatomically, the orbicularis oculi muscle is generally larger and thicker in elderly males than in females [28]. This necessitates gender-specific adjustments to BoNTA injection patterns and doses that may be needed to achieve the best results. Following this context, the panel also generally suggested that older male patients tend to require a higher dosage of BoNTA compared to female patients.

The “2-line injection” approach: Among Asian individuals, the distance from the lateral canthus to the lateral border of the orbicularis oculi muscle averages approximately 3.1 cm, but this measurement exhibits substantial interindividual variability [23]. The panel acknowledged the variability in aging and wrinkle severity among patients and recommended increasing the number of injection sites for older individuals to address wrinkles that extend with age. One example of such variation is a “2-line injection technique”, which involves administering additional injections further along the orbicularis oculi muscle to target wrinkles that extend beyond the typical treatment area or are associated with specific muscle movement patterns [26]. While a precise dosage for this technique was not established, the panel generally suggested that the total dosage should be comparable to or 2.5 U to 5 U higher than the classic three-point injection approach, with adjustments made based on the patient’s response and desired aesthetic outcome. This injection technique is depicted in Figure 6B.

Injection near the lateral canthus: Some experts presented a technique using a single, intradermal injection point near the lateral canthus with a very low dose (approximately 1 U) to address fine lines in this area. However, this technique should be cautiously approached due to the risk of the toxin spreading to nearby orbital muscles, potentially leading to eyelid ptosis or diplopia [29]. The panel was generally against the injection of BoNTA near the lateral canthus and emphasized careful palpation to identify the appropriate injection site and ensure adequate distance from the orbital septum.

Lower eyelid wrinkles: The palpebral portion of the orbicularis oculi muscle is subdivided into preseptal and pretarsal parts, located in the superficial layer of the orbital septum and tarsal plate. This portion involuntarily closes the eyelids when blinking, producing fine wrinkles in the lower eyelid skin [23]. A small amount, preferably 1 U, of BoNTA can be injected directly into the dermis of the pretarsal part to address fine wrinkles under the eyes (Figure 6B). Caution is required to avoid weakening the essential muscle functions, which could lead to complications such as lid edema, flattening of the pretarsal roll, lower eyelid fat bulging, or even scleral show [30].

Other considerations: Experts suggested that injectors should exercise caution when treating patients with asymmetric eyebrow height or narrow palpebral fissures, as BoNTA injection in the orbicularis oculi muscle may exacerbate these existing conditions. Additionally, a cautious dosing approach was recommended for individuals with deep-set eyes or thin skin to prevent excessive weakening of the orbicularis oculi muscle, which could lead to complications such as lagophthalmos or impaired eyelid function.

### 2.4. Dynamic Nasal Wrinkles (“Bunny Lines”)

**Figure 7:**
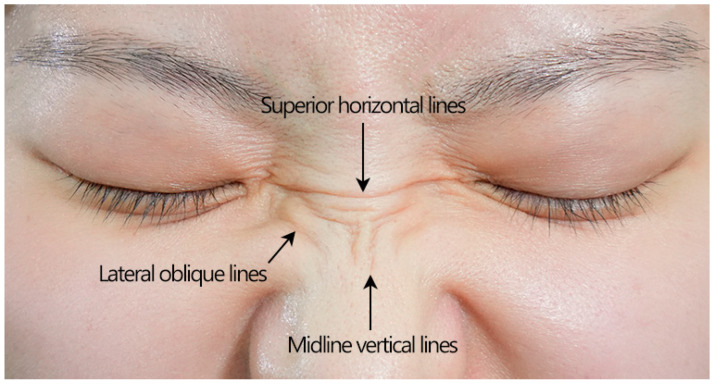
Key components of nasal dynamic wrinkles.

Dynamic nasal wrinkles, commonly referred to as “bunny lines”, are vertical and diagonal creases primarily attributed to the contractions of the nasalis muscle [31]. According to a recent analysis by Yi et al. [32], the dynamic wrinkles of the nasal skin can be attributed to three main components: (1) midline vertical lines, (2) lateral oblique lines, and (3) horizontal radix lines (Figure 7). While BoNTA cannot eliminate these nasal wrinkles, it can effectively reduce their appearance by targeting the specific muscles responsible for their formation.

#### 2.4.1. Anatomy and Patient Selection

Three principal muscles that form nasal wrinkles include the nasalis, procerus, and levator labii superioris alaeque nasi (LLSAN). The nasalis muscle has two parts: the transverse and the alar. The transverse part is triangular, originating from the maxillary canine fossa and inserting into the lateral cartilage of the nose, which wrinkles the nose horizontally. However, other nearby muscles, including the LLSAN, the procerus, and even the medial aspect of the orbicularis oculi muscle, are also involved and should be considered when treating nasal wrinkles [32]. The LLSAN is a long muscle that originates from the maxillary frontal process and inserts into the nasal ala and upper lip. The oblique component of the dynamic nasal wrinkles is primarily caused by the LLSAN, which elevates the upper lip, and partly by the medial muscular band of the orbicularis oculi. The procerus muscle pulls down the medial portion of the eyebrow while creating a transverse wrinkle between the glabella and the sellion.

Patient selection: Individuals with dynamic wrinkles on the nasal dorsum, particularly those exhibiting “bunny lines” or oblique nose furrows, are appropriate candidates for treatment. Generally, younger patients demonstrate a more favorable response to intervention.

#### 2.4.2. Recommended Standard Injection Technique 

**Figure 8:**
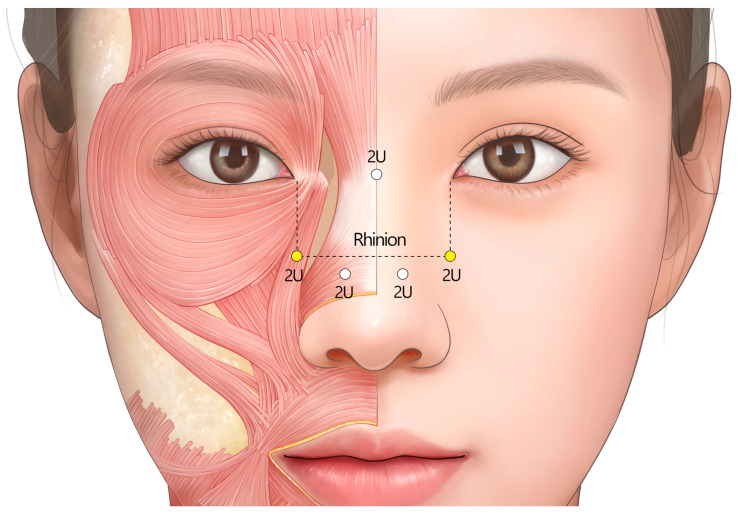
The recommended injection technique for dynamic nasal wrinkles: 2 U for horizontal radix lines to target the procerus; 2 U (×2) for midline vertical lines to target the transversal nasalis; and 2 U (×2) for the lateral oblique lines, targeting the levator labii superioris alaeque nasi muscle. U = unit(s); white circle = intradermal injection; yellow circle = subcutaneous injection.

To address midline vertical wrinkles, a dose of 2 U is injected into the superior ala of the nose on both sides, with the recommended injection point being the midpoint between the rhinion and the medial end of the supra-alar crease, targeting the transverse part of the nasalis muscle. For lateral oblique lines, 2 U of BoNTA is injected into the upper part of the LLSAN muscle on each side, with the recommended injection point being the crossing point of the horizontal line at the level of the rhinion and the vertical line at the level of the medial canthus. For horizontal radix lines, 2 U of BoNTA is injected into the nasal dorsum. The injection point is between the glabella and the sellion to target the procerus muscle. The specific total dosage may vary considerably depending on the chosen injection approach (Figure 8).

#### 2.4.3. Changes from the 2013 Guideline

The updated consensus emphasizes the roles of the procerus and LLSAN muscles, in addition to the transverse portion of the nasalis muscle, in the development of dynamic nasal wrinkles. The updated recommendation involves at least four injection points to provide a more comprehensive and effective treatment. Furthermore, the recommended treatment dosages have been refined better to reflect the varied contributions of these individual muscle groups.

#### 2.4.4. Expert Opinions

Precise targeting of the nasal muscles: In the lateral aspect of the lower nasal area, cadaver dissections consistently identify a triangular area that is uncovered by muscular structures, surrounded by the borders of the procerus, nasalis, orbicularis oculi, and LLSAN muscles [31]. This finding suggests that BoNTA injections on the oblique nasal lines in situ may inadvertently target connective tissue rather than the underlying muscles, frequently leading to suboptimal clinical results. It highlights the importance of precisely targeting the responsible muscles with BoNTA injections while avoiding unnecessary toxin diffusion into areas lacking muscle tissue. Accordingly, the panel recommended that BoNTA injections should ideally target the contributing muscles, such as the nasalis and LLSAN, rather than relying solely on the appearance of the external nasal wrinkles.

Minimizing the toxin spread: The panel emphasized the need for gentle and slow injection to prevent the spread of BoNTA to nearby tissues. This is crucial when treating nasal wrinkles, as unintended paralysis of the inferior or medial rectus muscles could lead to significant problems, such as diplopia [33]. Many panel members recommended manually blocking the inner boundary of the orbital rim during injection to prevent the spread of BoNTA. The panel also cautioned that injecting into the LLSAN could alter smile dynamics. Such injections may reduce the ability to elevate the upper lip, potentially leading to a less expressive or asymmetric smile.

### 2.5. Nasal Flaring and Nasal Tip Ptosis

The cosmetic use of BoNTA has recently been the subject of clinical interest and investigation for addressing nasal flaring. Nasal flaring, characterized by widening the nasal alae during facial expressions, can result in an aesthetically undesirable appearance. Nasal tip ptosis, the downward displacement of the nasal tip, represents another cosmetic concern that may be alleviated by applying BoNTA [34].

#### 2.5.1. Anatomy and Patient Selection

The dilator naris muscle is the primary contributor to nasal flaring, as it is responsible for widening the nostrils. Additionally, the zygomaticus minor muscle pulls the nostrils laterally, further contributing to nostril widening. The depressor septi nasi (DSN) is the primary driver of nasal tip ptosis. This muscle originates from the orbicularis oris and the periosteum superior to the central and lateral incisors, penetrates the nasal septum, and depresses the septum and the posterior portion of the nasal ala [35]. While the DSN is primarily involved in nasal tip depression, its downward pull can also indirectly lead to a wider nostril appearance.

Patient selection: Appropriate candidates for the treatment include individuals with prominent nasal flaring, especially during smiling, and those with a mildly drooping nasal tip seeking subtle aesthetic improvement.

#### 2.5.2. Recommended Standard Injection Technique (Figure 9)

Nasal flaring: The primary injection technique for addressing nasal flaring involves direct injection of 2 U of BoNTA into the dilator naris muscle, which is the primary driver of nostril widening or flaring. By placing an additional 2 U injection at the insertion point of the zygomaticus minor, its lateral pull on the nostrils is reduced, further minimizing flaring. Additional injection into the DSN to release the downward pull on the nasal tip can indirectly contribute to improving the appearance of the nostril widening.

**Figure 9:**
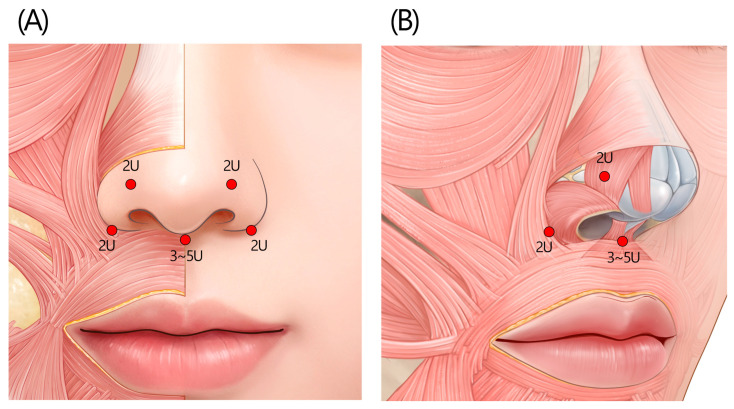
The recommended standard injection technique for nasal flaring and nasal tip ptosis. (**A**) Frontal view. (**B**) Oblique view. U = unit(s); red circle = intramuscular injection.

Nasal tip ptosis: The primary treatment approach for nasal tip ptosis is injecting 3–5 U of BoNTA into the DSN. A deep intramuscular injection is administered at the base of the columella, targeting the muscle’s attachment point to maximize the lifting effect. The effect may not be as dramatic in Korean patients as in Caucasian patients.

#### 2.5.3. Changes from the 2013 Guideline

The updated consensus guidelines recommend a more comprehensive approach to addressing nasal flaring, compared to the straightforward injection technique suggested in the 2013 guideline. In addition to the dilator naris, the panel recommended adjuvant injection of the zygomaticus minor muscle to address lateral nostril widening. For nasal tip ptosis, the panel maintained the recommendation of targeting the DSN.

#### 2.5.4. Expert Opinions

Patient Selection: Ideal candidates for BoNTA injections to address nasal flaring are individuals with a consistently elevated baseline tone in the dilator naris muscle, resulting in a persistently flared nasal appearance. In contrast, individuals with dynamic nasal flaring, where the flaring is observed only during specific facial expressions, may experience less pronounced or shorter-lasting treatment effects.

Dosage considerations: The dilator naris muscle plays a vital role in preventing the collapse of the nostrils during inhalation, which requires the muscle to possess relatively high contractile power despite its small size [36]. This helps explain why higher doses of BoNTA than expected are often needed to achieve a noticeable weakening and reduction in nasal flaring. Likewise, a relatively higher BoNTA dose is typically employed for nasal tip support to ensure sufficient weakening of the DSN to achieve a noticeable nasal tip lift.

### 2.6. Excessive Gingival Smile (“Gummy Smile”)

Some individuals reveal excessive upper gum mucosa when they smile or laugh, which can cause considerable embarrassment. As a result, they often try to smile only partially when being photographed or during social interactions, leading to a certain amount of anxiety, especially for those who are more self-conscious [37]. BoNTA can effectively address excessive gingival display during smiling.

#### 2.6.1. Anatomy and Patient Selection

The levator labii superioris (LLS), LLSAN, and zygomaticus minor contribute to the degree of lip elevation during a smile. The LLS originates from the orbital rim of the maxilla and inserts into the upper lip. In contrast, the LLSAN originates from the frontal process of the maxilla and inserts into the upper lip and nasal ala. The zygomaticus minor originates from the zygomatic bone and inserts into the upper lip skin [38]. The LLSAN muscle is generally considered the primary target for BoNTA injection in treating this condition [39]. However, some researchers suggest targeting the LLS and zygomaticus minor muscles together to enhance clinical effectiveness [40].

Patient selection: Appropriate candidates for treatment are individuals with moderate to severe excessive gingival display, exhibiting 3–5 mm of exposed gum when smiling due to overactive upper lip muscles.

#### 2.6.2. Recommended Standard Injection Technique

**Figure 10:**
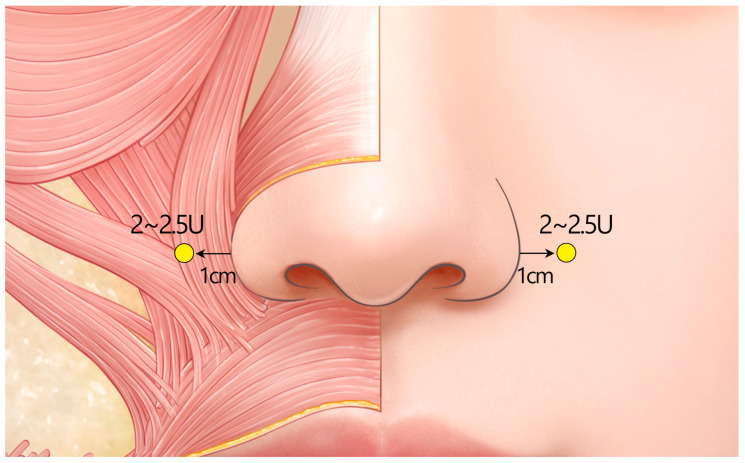
The recommended standard injection technique for the treatment of excessive gingival smile. Injections can be made subcutaneously or intramuscularly. Too deep intramuscular injections may result in the toxin deposited in the deep medial cheek fat, potentially reducing its efficacy. U = unit(s); yellow circle = subcutaneous injection.

Inject 2–2.5 U of BoNTA at the point 1 cm lateral to the outermost part of the alar crease (Figure 10). The recommended injection plane is the deep subcutaneous layer.

#### 2.6.3. Changes from the 2013 Guideline

The panel reaffirmed the central role of the LLSAN in gummy smile treatment while also acknowledging the potential contribution of other adjacent muscles. The recommended injection point and depth remain unchanged from the previous guideline.

#### 2.6.4. Expert Opinions

Patient selection: Patients with mild to moderate gummy smiles, characterized by excessive gingival display without significant skeletal discrepancies, are generally considered ideal candidates for BoNTA treatment. In severe cases, surgical corrections might be necessary to address the underlying skeletal issues contributing to the gummy smile. The expert panel advised careful consideration when treating patients with an elongated philtrum since BoNTA injections into upper lip elevators may slightly lengthen the cutaneous upper lip. This side effect could be exaggerated in individuals with a pre-existing long philtrum, potentially resulting in an old, sad-looking appearance.

Distance from the alar base: The panel discussed the optimal distance from the alar base for BoNTA injections to treat a gummy smile. Most panelists generally advocated maintaining a 1 cm distance from the alar base as the standard recommendation. This approach was deemed more effective for treating severe gummy smiles with significant gingival exposure, as it targets a broader range of muscles involved in upper lip elevation. However, some experts recommended injecting closer to the alar base, as this allows for more precise targeting of the LLSAN, potentially reducing the risk of affecting nearby muscles. This approach was considered suitable for milder cases, where weakening the LLSAN alone may be sufficient.

Additional injection points: The expert panel further discussed the potential benefits of incorporating additional injections into the LLS and DSN to achieve a more harmonious and balanced outcome in treating gummy smiles. Nevertheless, opposing viewpoints highlighted that the potential risks associated with injecting these additional muscles may outweigh the benefits, particularly for less experienced injectors. The panel concluded that the decision to include additional injection points should be based on the specific pattern and severity of the gummy smile presentation and a thorough assessment of the potential risks and benefits for the individual patient.

Injection depth: Some panel members favored intramuscular injections, highlighting that a deep injection helps ensure the toxin effectively reaches the target muscle fibers, particularly for deeper muscles like the LLSAN. Other members highlighted the technical challenges of intramuscular injections in the perioral region, where injecting too deeply could lead to the toxin being deposited in the deep medial cheek fat pad rather than the intended muscle, potentially reducing its efficacy. The panel generally agreed that the location of the injection point is more important than the injection depth, whether the injection is made subcutaneously or intramuscularly, to maximize the diffusion of the toxin into the target muscle.

### 2.7. Downturned Mouth

Oral commissure drooping, characterized by a downward turn of the mouth corners, can contribute to an aged or unhappy facial appearance [41]. A downturned mouth is a particular concern for many Koreans, as the lower position of the modiolus in Asian individuals can exacerbate the appearance of lip corner drooping [42]. In Korea, this condition is commonly addressed through BoNTA injection [1], which relaxes the underlying depressor anguli oris (DAO) muscle responsible for this undesirable characteristic.

#### 2.7.1. Anatomy and Patient Selection

The DAO is a triangular facial muscle originating from the mental tubercle. It forms an oblique line below and lateral to the depressor labii inferioris (DLI), converging to the modiolus and intertwining with the orbicularis oris and risorius muscles. The inferior and medial borders of the DAO are located approximately 1.5 cm lateral to the mandibular symphysis. This muscle acts to pull the corners of the mouth inferiorly. A recent cadaver study from Korea found the DAO to be located at a depth of about 3 mm from the skin surface [43].

Patient selection: The ideal candidates for treatment are individuals who exhibit visible muscle activity in the DAO region during facial expressions. Patients without significant skin laxity in the treatment area are suitable for this procedure.

#### 2.7.2. Recommended Standard Injection Technique

**Figure 11:**
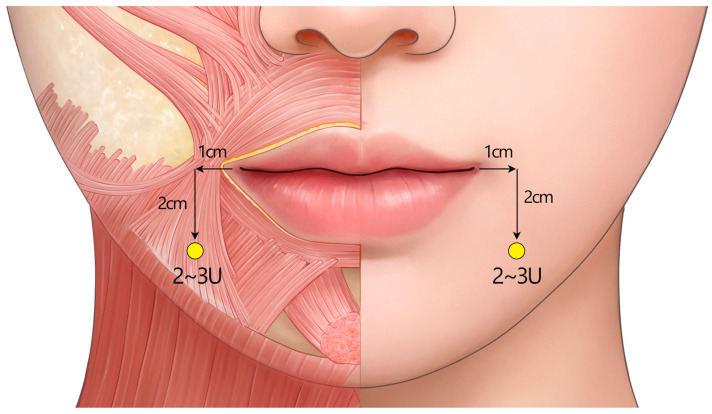
The recommended standard injection technique for mouth corner drooping. Excessive dosing or injecting too superiorly should be avoided since it can increase the likelihood of the toxin diffusing to the adjacent depressor labii inferioris muscle, which may result in lip asymmetry. U = unit(s); yellow circle = subcutaneous injection.

Measure 1 cm laterally from the corner of the mouth (chelion) and then 2 cm inferiorly from that point (Figure 11). This will target the lower part of the muscle belly of the DAO. Inject 2–3 U of BoNTA on each side subcutaneously.

#### 2.7.3. Changes from the 2013 Guideline

The 2024 guideline maintains the recommended dosage but suggests a slightly lower positioning of the injection sites compared to the previous suggestion. Notably, based on a recent cadaver study in Korea [43], the 2024 panel recommended administering the toxin superficially within the subcutaneous tissue without penetrating deeper structures, as opposed to the previously suggested intramuscular injection technique. The background of these changes is the prioritization of patient safety.

#### 2.7.4. Expert Opinions

Safety precautions: The expert panel consistently emphasized the importance of avoiding unintended effects on adjacent perioral muscles when targeting the DAO. Clinicians must possess comprehensive knowledge of the perioral anatomy and the potential risks associated with BoNTA injections, particularly lip asymmetry. Precise injection techniques, appropriate dosage selection, and meticulous attention to detail are essential for minimizing the risk of complications.

Individualized adjustment: While the landmark-based approach provides a starting point, the panel emphasizes the importance of individualizing the injection point. Injectors are strongly encouraged to observe the patient’s dynamic facial expressions, particularly during frowning or pouting, to identify the area of maximum DAO contraction.

Dosage: The expert panel recommends a dosage range of 2–3 U of BoNTA for each DAO. This range balances efficacy and safety, minimizing the risk of overtreatment. The panel advocates a conservative approach, starting with 2 U for the initial injection. This allows for assessing the patient’s response and minimizing the risk of unwanted side effects. A follow-up injection can be administered after 2 weeks to enhance the results. Injecting more than 4 U for each DAO is generally discouraged due to the increased risk of toxin diffusion to adjacent muscles, particularly the DLI, which could lead to muscle weakness, asymmetry, and an unnatural appearance.

Platysma injections and combination with fillers: While 3 U of BoNTA injection has been demonstrated to increase the height of the DAO significantly, it may not always correlate with patient satisfaction when only the DAO is injected [44]. The panel suggested combining DAO injections with adjuvant treatments could enhance clinical efficacy and achieve more comprehensive and satisfactory outcomes. They recommended considering injections into the platysma muscle when additional correction is needed, as this muscle also contributes to the downward pull of the mouth corners. The panel notes that the dosage for platysma injections can be more flexible than DAO injections. The dosage should be determined based on the degree of platysmal activity and the desired aesthetic outcome. In addition, combining BoNTA injections and dermal fillers can provide more comprehensive results for addressing downturned mouth and marionette lines. BoNTA relaxes the underlying muscles, while fillers add volume and smooth out static wrinkles. This dual approach can enhance overall facial aesthetics and promote a more balanced, natural appearance.

### 2.8. Dimpled Chin (“Cobblestone Chin”)

BoNTA is commonly utilized to target the mentalis muscle and mitigate the appearance of a “cobblestone” or dimpled chin. This condition arises from hyperactivity of the mentalis muscle, as its fibers insert into the dermis of the chin [45]. Given the mentalis muscle’s role in elevating the chin and lower lip, its hyperactivity manifests as a shortened chin appearance. BoNTA injections relax the mentalis muscle, thereby protruding the chin and producing a more refined, aesthetically pleasing chin contour.

#### 2.8.1. Anatomy and Patient Selection

The mentalis muscle originates from the anterior aspect of the mandible below the lateral incisors and inserts into the dermal layer of the chin. The muscle belly is typically divided into two parts with intervening fatty tissue. The mentalis muscle comprises medial, lateral, superior, and inferior fibers. The medial fibers have a dome-like descent, while the lateral fibers attach obliquely to the skin. The superior fibers interdigitate with the inferior border of the orbicularis oris muscle, assisting in lip elevation. The attachment of the muscle fibers to the skin accounts for the formation of dimples or indentations upon muscle contraction [45].

Patient selection: Individuals who exhibit visible contraction or hyperactivity of the mentalis muscle while pursing the lower lip are suitable candidates for this treatment. This can be assessed by observing the patient’s dynamic facial movements and noticeable dimpling or indentation in the chin area.

#### 2.8.2. Recommended Standard Injection Technique

**Figure 12:**
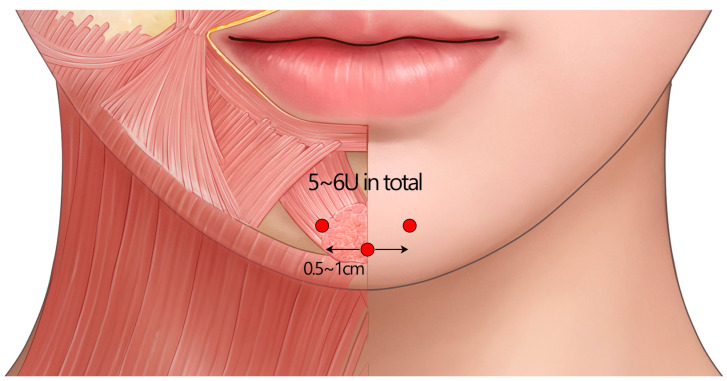
The recommended standard injection technique to treat a dimpled chin appearance, involving a deep, intramuscular injection delivered perpendicular to the skin surface. U = unit(s); red circle = intramuscular injection.

The recommended technique involves three injection points: one at the midline of the chin and two additional injections on each side, positioned 0.5–1 cm lateral to the midline. This strategy specifically targets the most prominent region of the external chin at the level of the pogonion. The recommended approach is a deep, intramuscular injection delivered perpendicular to the skin surface. The typical total dose is 5–6 U, distributed evenly across the three injection sites (Figure 12).

#### 2.8.3. Changes from the 2013 Guideline

The 2024 guideline retains the general principles of the previous recommendations but provides more conservative guidance on injection dosage. The maximal total dosage has been reduced significantly from 15 U to 5–6 U. In addition, the 2024 guideline emphasizes the importance of administering BoNTA injections directly into the mentalis muscle using the intramuscular approach, moving away from the previously recommended optional intradermal injections.

#### 2.8.4. Expert Opinions

Labiomental crease: The panel discussed the inclusion of the labiomental crease, the groove between the lower lip and chin, in the injection area. Concerns were raised about the potential diffusion of the neurotoxin beyond the intended treatment area, which could affect the orbicularis oris and the DLI muscles. As the orbicularis oris is responsible for general lip movement and the DLI lowers the lower lip, unintended paralysis of these muscles could lead to side effects like mouth drooping, speech difficulties, or asymmetry. To mitigate these risks, the panel recommended avoiding injections into the labiomental crease area. Targeting the inferior part of the chin directly and focusing on the mentalis muscle belly minimizes the risk of toxin diffusion while effectively addressing the cobblestone appearance.

Deeper injections: In Korea, intradermal injections have become more common in addressing superficial irregularities of the chin. However, the panel reached a consensus favoring intramuscular injections, regardless of the severity of the cobblestone appearance. This shift is grounded in the understanding that directly targeting the mentalis muscle bellies yields more predictable and long-lasting results. While intradermal injections might improve surface irregularities, they may not effectively address the root cause of the issue―the mentalis muscle contraction itself.

Post-surgical patients: Mentalis muscle function and lower lip position alterations can occur in patients who have undergone mandible surgeries. BoNTA injections in these patients require careful consideration of the altered anatomy and muscle dynamics. In such cases, the injector should thoroughly assess the patient’s mentalis muscle activation pattern and appropriately adjust the injection sites and depths, deviating from standard protocols when necessary.

Adding volumes: A combined approach involving dermal fillers and BoNTA benefits patients with retruded chins. Fillers can address underlying skeletal deficiencies in these patients, complemented by BoNTA injections to relax the musculature. This integrated approach optimizes aesthetic outcomes by addressing the structural and functional components of the soft tissue framework. According to a recent randomized, controlled study, the combined therapy achieved comparable improvements in chin retrusion measurements while requiring a lower overall filler volume, indicating superior treatment efficacy for patients with chin retrusion [46]. The panel recommended waiting at least one week after the BoNTA injection to assess whether the clinical results are sufficient with only the toxin injection. The decision to use supplemental filler injection and the required filler volume is left to the discretion of the injector and the patient.

### 2.9. Decision-Making Framework for BoNTA Dosage

The panel suggested using a decision-making framework (Table 1) to determine the appropriate BoNTA dosage for targeting facial expression muscles. This framework considers various patient-specific factors to ensure a tailored treatment approach based on the panel’s experience.

## 3. Conclusions

The updated consensus guideline provides a comprehensive update from Korea on the current recommendations, changes, and comments for using BoNTA to improve various cosmetic concerns caused by facial expression muscles. It also reflects on an evolution in the treatment patterns observed in Korea over the past decade, with some recommendations modified, others excluded, and a subset still being followed. However, it is acknowledged that there is ample room for improvement, as not all recommendations in the present guidelines are directly supported by strong evidence. A primary limitation of this paper is the lack of high-quality scientific evidence supporting the recommendations and suggestions presented. The consensus recommendations presented are primarily based on expert opinions and consensus, clinical case series, and anatomical studies, which may largely be evaluated as “weak” or “conditional” according to the Grading of Recommendations Assessment, Development, and Evaluation (GRADE) framework for practice guidelines [47], giving the paper a descriptive and qualitative nature. Future guideline development in this field may benefit from leveraging current consensus recommendations, including the present one, insights from domestic and international round-table discussions, and evidence-based systematic reviews. Additionally, further research and prospective studies are needed to strengthen the scientific foundation of these guidelines and ensure they are aligned with the latest advancements in aesthetic medicine.

## 4. Materials and Methods

On 4 February 2024, a panel of ten multidisciplinary Korean experts in dermatology, plastic surgery, aesthetic medicine, and clinical anatomy met to update and formulate consensus recommendations for cosmetic uses of BoNTA. Prior to the convening of the expert panel, the members reviewed previously published consensus guidelines or recommendation papers on the facial cosmetic uses of BoNTA, including the 2013 Korean consensus guideline, which was developed by a group of dermatologists, some of whom were involved in the development of the current updated consensus paper. During the meeting, the panel thoroughly reviewed the standard adult Korean facial photos, pinpointed the preferred injection sites on detailed anatomical diagrams, and shared comprehensive recommendations on the most effective injection techniques and appropriate dosages to be utilized. Potential complications were also discussed, along with their prevention strategies, based on the panel’s professional experience and relevant scientific literature. The discussion topics were limited to cosmetic conditions associated with facial expression muscles. Recommendations excluded high-risk injection sites and applications requiring significant expertise to provide straightforward, practical advice for the safe and effective use of BoNTA, reflecting the best practices of average aesthetic healthcare professionals.

The recommendations were developed using a Delphi-based consensus framework [48]. The Delphi survey presented participants with five response options: (1) Agree, (2) Strongly Agree, (3) Neutral, (4) Disagree, and (5) Strongly Disagree. Participants selected one response and provided comments and feedback to facilitate discussion. Consensus was reached if at least 80% of participants chose either “Agree” and/or “Strongly Agree”, or “Disagree” and/or “Strongly Disagree”. Items that did not achieve consensus were omitted from the next round. However, if participants provided feedback regarding the clarity of the questions or their clinical relevance, the questions were rephrased for clarity and re-evaluated in the subsequent round. This process involved a total of four rounds of iterative discussion.

The suggested dosage guideline pertains to a novel BoNTA product (NEWLUX^®^: NUMECO, Seoul, Republic of Korea), which is used routinely by panel members in their current practice. The NEWLUX^®^ product has been demonstrated to be generally comparable to the BOTOX^®^ (Allergan, Irvine, CA, USA) formulation, with a 1:1 dose conversion ratio [49]. Table 2 summarizes the formulation details of NEWLUX and other widely used BoNTA products in the Korean market. The recommended injection techniques were illustrated by a skilled medical artist who specialized in clinical facial anatomy and had extensive experience in depicting the intricate musculature and structures of the human face. To further enhance the accuracy and reliability of the illustrations, they were meticulously reviewed by another clinical anatomist with expertise in the facial anatomy of adult Korean patients.

## Figures and Tables

**Table 1:** A structured decision-making algorithm for botulinum toxin dosage when targeting facial expression muscles.

Step 1: Patient Assessment
Age: Assess the patient’s age, as younger patients may have different muscle mass and skin elasticity compared to older patients. Gender: Consider gender differences in muscle mass; men may require higher doses than women due to greater muscle volume. Skin quality: Evaluate skin elasticity and texture to determine how well the skin might respond to treatment.
Step 2: Evaluate Muscle Activity
Muscle strength: Assess the strength and activity level of the targeted muscles during facial expressions. This can include observing the degree of contraction when the patient performs specific expressions. Muscle mass: Utilize tools such as the Glabellar Muscle Mass Scale to evaluate muscle mass and adjust dosing accordingly.
Step 3: Initial Dosage Recommendation
When treating areas like the glabella and chin, use the standard doses recommended in the guideline. For riskier areas such as the forehead and around the mouth, consider starting with slightly lower doses than those suggested in the guideline.
Step 4: Adjust Dosage Based on Patient Factors
Previous treatment response: Review the patient’s history with botulinum toxin. If they have had previous treatments, consider their response duration and any side effects experienced. Patient expectations: Discuss with patients their expectations regarding results. Adjust dosage if they desire a more subtle or more pronounced effect.
Step 5: Monitor and Follow-Up
Post-treatment assessment: Schedule follow-up appointments to assess muscle response and satisfaction with results. Adjust future dosages based on observed effects and patient feedback. Re-treatment protocol: Consider re-treating every 3–4 months, adjusting the dosage as needed based on previous outcomes. However, excessively frequent re-treatment with higher doses could theoretically increase the risk of antibody formation.

**Table 2:** Selected listing of botulinum neurotoxin type A formulations marketed in South Korea.

Trade Name	Manufacturer (Country of Origin)	*C. botulinum* (Type A) Strain	Finishing	Size	Units in Vial	Excipients
BOTOX^®^	Allergan (USAAllergan (Irvine, CA, USA))	Hall hyper	Vacuum dried powder	900 kDa	200/100/50 Allergan Units	HSA
Dysport^®^	Ipsen (Boulogne-Billancourt, France)	Hall ATCC3502	Lyophilized powder	400 kDa	500/125 Speywood Units	Lactose HSA
Xeomin^®^	Merz (Frankfurt am Main, Germany)	Hall ATCC3502	Lyophilized powder	150 kDa	200/100/50 Merz Units	Sucrose HSA
Neuronox^®^	Medy-Tox (Seoul, Republic of Korea)	Hall hyper	Lyophilized powder	900 kDa	200/150/100/50 Units	HSA
Coretox^®^	Medy-Tox (Seoul, Republic of Korea)	Hall hyper	Lyophilized powder	150 kDa	100 Units	Polysorbate 20 L-methionine
Innotox^®^	Medy-Tox (Seoul, Republic of Korea)	Hall hyper	Liquid	900 kDa	25 Units	Polysorbate 20 L-methionine
NEWLUX^®^	NUMECO (Seoul, Republic of Korea)	Hall hyper	Lyophilized powder	900 kDa	100 Units	HSA

C. = Clostridium; HSA = human serum albumin.

## Data Availability

The original contributions presented in this study are included in the article. Further inquiries can be directed to the corresponding author.
